# Lipolytic Enzymes with Hydrolytic and Esterification Activities Produced by Filamentous Fungi Isolated from Decomposition Leaves in an Aquatic Environment

**DOI:** 10.1155/2019/8182425

**Published:** 2019-06-02

**Authors:** D. B. Mendes, F. F. Da Silva, P. M. Guarda, A. F. Almeida, D. P. de Oliveira, P. B. Morais, E. A. Guarda

**Affiliations:** ^1^UFT, Brazil; ^2^UFT Campus Palmas, Brazil; ^3^UFT Campus Gurupi, Brazil; ^4^INPA, Brazil

## Abstract

Microbial lipases are prominent biocatalysts able to catalyze a wide variety of reactions in aqueous and nonaqueous media. In this work, filamentous fungi isolated from leaves decomposed in an aquatic environment were screened for lipase production with hydrolytic activity and esterification. Agar plates with Tween 20 and Rhodamine B were used for selection, while submerged cultures with olive oil were subsequently used to select 38 filamentous fungi.* Trichoderma harzianum*,* Fusarium solani*,* Trichoderma harzianum* F5, and* Penicillium *sp. F36 were grown in six different culture media.* F. solani* presented the highest lipase production (2.37 U/mL) with esterification activity of 0.07 U/mL using medium composed of (g.L^−1^) KH_2_PO_4_ 1.00, MgSO_4_ H_2_O 1.123, and CuSO_4_ 0.06. Supplementation of this culture medium with organic nitrogen sources increased lipase production by 461.3% using tryptone and by 419.4% using yeast extract. Among the vegetable oils from the Amazon region, degummed cotton oil induced lipase production up to 8.14 U/mL. The lipase produced by* F. solani* F61 has great potential to application in conventional processes and biodiesel production by transesterification of vegetable oils, as well as food industries in the production of fatty acid esters by hydrolysis and esterification.

## 1. Introduction

Lipases (triacylglycerol acyl hydrolase, E.C. 3.1.1.3) have emerged as key enzymes in swiftly growing biotechnology, owing to their multifaceted properties, which find usage in a wide array of industrial applications, such as food technology, detergent, chemical industry, and biomedical sciences [1]. These enzymes are able to catalyze many reactions on ester bonds with preference on water-insoluble substrates being activated when absorbed to an oil-water interface to generate free fatty acids, monoacylglycerols, diacylglycerols, and glycerol [2]. Lipases are organic solvent-tolerant which open up an infinity number of application for the chemical industry, as esterification reactions are used to produce food flavor, fragrance, cosmetic and pharmaceutical, as well as transesterification to produce biodiesel [3–5].

Lipase production has been developed mainly by submerged fermentation due to the dominated and developed engineering aspects; however, they can also be produced by solid-state cultivation [6, 7], in which insoluble substrates with low percentages of water in their composition are used [8].

There are several methods of selection of lipase-producing organisms, lipolysis being observed directly by changes in the appearance of the substrate [9], where lipase production is indicated by the formation of light halos around cultured colonies on agar plates containing tributyrin, triolein, and tweens [10]. The lipolytic activity in solid media can also be determined using dyes such as Rhodamine B [7] and Vitoria B blue [11], in which a drop in pH occurs due to the fatty acids released by the hydrolysis of the substrate, resulting in the formation of fluorescent halos around the colony [12]. The combination with Rhodamine B proved to be an efficient method in which this compound binds fatty acids and mono- and diglycerides and develops fluorescence under ultraviolet (UV) light. Thus, this technique is very convenient since Rhodamine B is quite selective and used for the selection of microorganisms producing lipase [13].

Lipases are inducible enzymes, i.e., produced by microorganisms in the presence of an inducer, which may be the substrate itself or the product of its hydrolysis. The inducer can be added to the culture medium to stimulate production [14], requiring sources of lipid carbon in the form of oils for higher enzyme yield [15]. The mechanisms that regulate lipase biosynthesis vary significantly among the different producing microorganisms. In some cases, lipase production appears to be constitutive and independent of the addition of lipid substrates in the culture medium [16]. However, their presence may increase the level of lipolytic activity. There are several reports of the use of tested vegetable oils to evaluate their induction power in the production of lipases by filamentous fungi, such as palm oil [17], canola oil, corn oil, sunflower oil [18], castor oil [19], and cotton oil [20].

The aims of this work were to select strains of filamentous fungi isolated from aquatic environments in tropical ecosystems, which are producers of lipolytic enzymes with hydrolytic activity and esterification, and to evaluate the nutritional conditions required to produce this enzyme under submerged conditions.

## 2. Material and Methods

### 2.1. Microorganisms

The filamentous fungi strains were isolated from decomposition leaves harvested in Buritizal stream located in -10 15' 51,91040” latitude and -48 06' 54,7474” longitude, Palmas city, State Tocantins, Brazil. The microorganisms were cultured in Potato Dextrose Agar (PDA) media (potato extract: 4.0 g; dextrose: 20.0 g; agar: 15.0 g), supplemented with 0.1 *μ*g/mL chloramphenicol, and incubated at 28°C for 7 days.

### 2.2. Molecular Identification

Total DNA from the filamentous fungal strains were extracted from cultures grown on PDA media using the IllustraTM Nucleon PhytoPure, plant and fungal DNA extraction kits (GE Healthcare, Amersham, England). The amplified products were purified and the samples were then sent to the Laboratory of Polar Microbiology and Tropical Connections of the Federal University of Minas Gerais (UFMG) and to the Laboratory of Cellular and Molecular Parasitology (LCPM), Oswaldo Cruz Foundation (FioCruz) Institute/René Rachou. The sequencing products of both DNA strands were contiguously grouped, aligned, and corrected using the Geneious 6.1.8 software [27]. A comparative identity search of the nucleotide sequences obtained from the isolates was performed using the BLAST (Basic Local Alignment Search) tool [28] on NCBI (National Center for Biotechnology Information). Identity ≥ 99% confirmed the identity of the species.

### 2.3. Screening of Filamentous Fungi for Lipolytic Enzyme Production

*Initial screening for lipolytic enzyme production *(*qualitative method*): Lipase-producing filamentous fungal strains were screened on PDA plates supplemented with 1.0% (w/w) olive oil and Rhodamine B (0.01%, ww). Lipolytic activity was detected by the appearance of orange fluorescence halos around the colonies, under UV light at 350 nm [29]. A second growth medium was carried out according to Hankin and Anagsnostakis (1975) composed of (g/L) peptone, 10; NaCl, 5; CaCl_2_*∗*2H_2_O, 0.1, agar, 20, supplemented with 1.0% (w/v) Tween 20. The final pH was adjusted to 7.4 and the medium was autoclaved at 121°C, for 20 min. The lipolytic activity was indicated by appearance of opaque halos around the colonies due to calcium salts precipitation. Cultures were incubated at 28°C and examined daily for 7 days. The enzymatic activity was determined using an enzymatic index (IE), where the relation of the mean diameter of the degradation halo and the mean diameter of the colony is evaluated [10]. In this work, due to the use of two applied techniques in the initial selection, it was not possible to calculate the enzymatic index by measuring the fluorescence halo or opaque area around the colony. Therefore, each strain was assigned a positive (+) or negative (-) for the presence/absence of activity, respectively, expressed under various conditions using different media. This assigned signal was used as our enzymatic activity.

*Second screening of lipolytic enzyme production *(*quantitative method*): Liquid medium was prepared using Vogel's medium [30]. Trace elements solution (solution A) was prepared containing (g/L) citric acid*∗*H_2_O, 50, ZnSO_4_*∗*7H_2_O, 50, Fe(NH_4_)_2_(SO_4_)_2_*∗*6H_2_O, 10; CuSO_4_*∗*5H_2_O, 2.5; MnSO_4_*∗*H_2_O, 0.05; H_3_BO_3_, 0.05; Na_2_MoO_4_*∗*2H_2_O, 0,05. Salt solution (solution B) was prepared containing (g/L) sodium citrate*∗*5H_2_O, 150; KH_2_PO_4_, 250; MgSO_4_*∗*7H_2_O, 10; CaCl_2_*∗*2H_2_O, 5 and biotin solution (0.1 mg/mL) 5 mL; 0.2 mL chloroform. The saline solution (solution B) was maintained as a stock solution. In one liter of the trace elements solution (solution A), 5 mL of saline solution B, 5 mL of the biotin solution, and 2 mL of chloroform as antimicrobial agent were added to make up the Vogel media. The solutions were maintained at 4°C. The preparation of the medium consisted of a 50-fold dilution of solution A in distilled water, substituting glucose for 1.0% olive oil, and 0.2% yeast extract and the final pH was adjusted to 6.0.

### 2.4. Enzyme Assays

#### 2.4.1. Determination of Hydrolytic Activity

Lipase activity was assayed with *ρ*-nitrophenyl-palmitate (p*-*NPP) as substrate [31]. p*-*NPP was firstly solubilized in 0.5 mL of dimethyl sulfoxide and then diluted to final concentration at 50 mM with 50 mM sodium phosphate buffer pH 7.0, containing 0.5 % Triton X-100. Lipase activity was determined by following the hydrolysis of* p*-NPP at 37°C. The reaction was stopped at different intervals by heat shock (90°C, 1min), followed by the addition of 1 mL saturated sodium tetraborate solution. The calibration curve was prepared using pNP as standard. The* p*-nitrophenol formed was measured spectrophotometrically at 405 nm. The molar extinction coefficient for *ρ*-nitrophenol (p-NP) at 405 nm is 1.8 x 10^4^ M/cm^−1^. Controls were prepared without enzyme. One unit of enzyme activity was defined as the amount of enzyme that releases 1 *μ*mol of p-NP per min. Results were presented as total units or specific activity in units by milligram of protein.

#### 2.4.2. Determination of Esterification Activity

The esterification activity (EA) catalyzed by the crude enzymatic extracts was followed by modified titrimetric assays [32]. The esterification activity of crude enzymatic extracts was measured by the consumption of acid in the esterification reaction with ethanol and oleic acid (1:1 equimolar ratio). Experiments were carried out in Erlenmeyer flasks (125 mL) containing 5 mL of reaction medium. The reaction was initiated by the addition of 1 mL of the enzymatic extract. Experimentally, a control assay (blank) was consistently performed. After incubation in shaker for 120 min at 40°C and 180 rpm, the content of fatty acids remaining in the aliquot was extracted by the addition of 15 mL of an acetone/ethanol solution (1:1, v/v). The amount of oleic acid was then titrated with 0.1 M NaOH up to pH 11. One unit of lipase activity was defined as the amount of dry enzyme preparation necessary for the consumption of 1 *μ*mol.min^−1^ of acid under assay conditions. All assays were performed in duplicate, and the results are expressed in terms of units per gram of dry substrate and calculated as follows:

(1) $$\begin{array}{c} EA=\frac{\left(Va-Vb\right)\;\;x\;\;M\;\;x\;\;100}{E\;\;x\;\;t} \end{array}$$

where Va is volume of NaOH used after 120 min (mL); Vb is volume of NaOH used at zero time of reaction (mL); M is molarity of NaOH solution; t is time (min).

### 2.5. Enzyme Production with Different Culture Media Compositions

Cultures were grown in triplicate in Erlenmeyer flasks (250 mL) containing 25 mL of respective medium described in Table 1, supplemented with 1.0% (w/v) olive oil and 0.2% (w/v) yeast extract. Media were autoclaved at 121°C for 20 min. Inocula were prepared using 5-day-old cultures. Medium was inoculated with 1 mL conidia suspension (10^7^ spores per mL) and incubated at 28°C, 180 rpm for 7 days. Biomass was separated from the fermentation broth by filtration (membrane cellulose acetate 0.45 *μ*m cut-off) and dried at 105°C until constant weight. Broth was used for hydrolytic and esterification activity assays in triplicate.

### 2.6. Effect of Nitrogen and Carbon Sources on Hydrolytic Enzymes Production

Nitrogen sources were evaluated using organic sources (peptone, yeast extract, corn steep liquor, tryptone, and urea) and inorganic sources (ammonium sulfate and ammonium chloride) at different concentrations (0.2, 1.0, and 2.0%, w/v). Control culture was carried out without nitrogen sources.

Hydrophobic carbon sources such as castor oil (*Ricinus communis*), pequi oil (*Caryocar brasiliense*), babassu oil (*Attalea speciosa*), copaiba oil (*Copaifera langsdorffii*), buriti oil (*Mauritia flexuosa*), degummed soybean oil (*Glycine max*), degummed cotton oil (*Gossypium hirsutum*), and soybean oil fried at concentrations of 1.0% (w/v) were used in place of olive oil. Control culture was carried without lipidic carbon sources. The carbon sources used in this work were chosen due to the chemical diversity of these oils; in addition, they are sources that belong to Legal Amazonian.

### 2.7. Time-Course of Cultivation

Time-course of cultivation of filamentous fungi was carried out in Erlenmeyer flasks (250 mL) containing 25 mL of selected culture medium, carbon, and nitrogen sources and cultivated for 10 days, at 28°C and 180 rpm. At this stage, the samples were collected periodically and biomass and enzyme production were analyzed. Cultures were inoculated individually in each flask using 1 mL conidia suspension (10^7^ conidia per mL).

### 2.8. Statistical Analyses and Determination of Pearson Correlation

For the statistical analysis of the data obtained, the software Sisvar version 5.7 was used. The results were analyzed through Scott-Knott test for comparison of means at the level of significance 0.05. The Pearson correlation coefficient (r) varies from -1 to 1 [33]. The Pearson correlation coefficient in this work was calculated to determine the existence of correlation between biomass production and the production of enzymes with hydrolytic activity. The “Online Correlation Coefficient Calculator” was used here and is available at http://www.sthda.com/english/rsthda/correlation.php.

## 3. Results and Discussion

### 3.1. Screening of Filamentous Fungi for Lipolytic Enzyme Production with Hydrolytic and Esterification Activities

Thirty-eight filamentous fungi isolated from decomposition leaves were evaluated for lipolytic enzymes in agar plates by enzymatic activity formed by Rhodamine B and calcium precipitation halos with Tween 20 (Table 2). Potential enzymes production by filamentous fungal strains also was evaluated in submerged cultivation and biomass. Hydrolytic activity and esterification activities were also evaluated (Table 3). Among fungal strains evaluated, 66.0% presented positive enzymatic activity in agar plates using olive oil or Tween 20 as carbon sources. Solid agar plate was indicated as a direct method to identify fungal growth as well as the ability to produce extracellular enzymes. Furthermore, it is a useful rapid method to evaluate individual fungi of genetic variants for either presence or absence of enzymes such as lipases [34]. Smitha et al. (2014) screened 181 marine fungi for their ability to secrete hydrolytic enzymes using nutrient agar supplemented with tributyrin as substrate. The authors observed that 60.2% of total fungi isolates presented lipolytic activity. The sensitivity of the agar plate method to identify active and true lipase is dependent on several factors, e.g., the microorganism's growth, production and diffusion of the enzymes, activity and specificity of the enzyme to substrate, and enzyme concentration. Other factors can also influence halo formation, such as medium composition, growth temperature, and chemical and biological environment [35–37]. On the other hand, the halo formation does not represent the volumetric enzyme production, and the additional experiment, such as submerged cultivation, is needed to identify the promising microorganisms for bioprocess steps (Colen, 2006). Winayanuwattikun et al. (2011) used the agar plate method to screen fungal lipase with activity on long-chain triacylglycerides. In this work, the authors selected 39.2% strains with fluorescence halos on palm oil hydrolysate, indicating the lipase production with specificity for long-chain fatty acids.

A second step was carried out under submerged cultivation to evaluate the lipase production by filamentous fungi that presented positive enzymatic activities on agar plates with olive oil as sole carbon source. Furthermore, the cultivation that presented hydrolytic activity on* p*-nitrophenyl palmitate was also evaluated for esterification activity and biomass production (Table 3). Twenty-five strains that presented enzymatic activity on agar plate also presented hydrolytic activity under submerged cultivation. The highest enzyme activity was observed with strain F61 (0.62 U/mL). Intermediary enzyme activity was observed with eight filamentous fungi (0.12 – 0.25 U/mL) and lowest enzyme activity (0.01 – 0.08 U/mL) was observed for sixteen filamentous fungal strains. Goldbeck et al. (2013) observed that, from 372 strains evaluated, 207 showed a hydrolysis halo around the colonies, representing 55.6% of the total. Almeida et al. (2013) evaluated the lipolytic enzyme production for 11 filamentous fungi and 8 yeast strains on agar plates, where 21% of the microorganisms presented potential to enzyme production. Among them, 63.0% presented lipase activity on* p*-NPP substrate when cultured in submerged conditions. Griebeler et al. (2011) observed that, among the 203 isolated strains, the filamentous fungi presented a higher potential for lipase production on agar plate and in solid-state cultivation; however, some microorganisms that presented with hydrolysis halos on agar plate with tributyrin as carbon source did not show hydrolytic activity when cultivated under solid-state conditions with soybean bran.

Among the fungal strains that produced hydrolytic activities, the esterification activity was observed in 36.0% of the total number of isolates. The highest esterification activity (0.11 U/mL) was observed with F36 strain, followed by F307 (0.10 U/mL), F5 and F61 (0.09 U/mL), and F37 (0.08 U/mL) (Table 3). Lipases in organic synthesis are very prominent and the screening of lipases that work adequately in organic medium for ester synthesis, converting triglycerides to alkyl esters and triacylglycerol interesterification, has tremendous application particularly in the food industry [34]. Cardenas et al. (2001) selected fungal lipase that worked well in organic media for the synthesis of heptyl oleate. They observed that* Monascus mucoroides, Fusarium poae, Fusarium solani*,* and Ovadendron sulphureo-ochraceum* showed a higher esterification activity, respectively. In this study, it was observed that lipases from* F. poae* and* F. solani*, which had showed a low hydrolytic activity, were very productive in the synthesis of heptyl oleate.

Biomass produced by filamentous fungi indicates that each isolated strain could grow on liquid medium supplemented with olive oil, and no correlation was found to exist between lipase and biomass production (*R*-Pearson=0.16) (Table 3). Maximal biomass production was observed for F24 strain (10.76 g/L). The F61 strain produced higher lipase activity (0.62 U/mL) and intermediary biomass (5.12 g/L) compared to the other fungal isolates. Messias et al. (2011) also observed no correlation between lipase and biomass production of* Botryosphaeria* isolates with different oils and glycerol carbon sources. The authors observed that* Botryosphaeria ribis* EC-01 grown in glycerol presented high lipase activity and less biomass when compared to the other vegetable oils evaluated.

### 3.2. Molecular Identification of Fungal Strains

The filamentous fungi that presented potential to produce lipase with hydrolytic and esterification activities in the initial stage of work were identified using morphological characteristics, based on shape of fructification body (F5 and F36) and molecular technique (F61, F3, F21, F37, F104, F116, F120, F125, F130, and F307), using ribosomal targets ITS-1 and ITS-4 (Table 4). The nuclear ribosomal internal transcribed spacer (ITS) region was used in this study for the molecular identification of the filamentous fungi, as it is generally accepted as the universal DNA barcode marker for fungi [38] and has often been used for the molecular identification of various species of fungi [39, 40]. Molecular identification of the ITS region showed high similarity (99%) with five genera (*Aspergillus, Chaetomium, Fusarium, Penicillium*, and* Trichoderma*), grouped into nine species, which are* Aspergillus calidoustus*,* Aspergillus oryzae, Aspergillus flavus* (n = 2 isolated),* Chaetomium aureum, Fusarium solani, Penicillium citrinum,* and* Trichoderma harzianum *(n = 2 isolated). One isolate showed high sequence similarity with an isolate identified only as* Aspergillus *sp. The genus* Fusarium* has been widely recognized as lipase producer in several studies [41–43], with the species* Fusarium solani* being among the well-known lipase producers. A BLAST search on the NCBI database (http://blast.ncbi.nlm.nih.gov/Blast.cgi) of the ITS-1 and ITS-4 sequenced fragment showed 99% sequence identity with* T. harzianum* for F37 and 307 strains and* F. solani* for F61. The strains F5 and F36 were identified as* Trichoderma *sp. F5 and* Penicillium *sp. F36, respectively, based on the morphological characteristics (colony appearance, color, and type of mycelium).

### 3.3. Effect of Culture Media Composition on Enzyme Production

In this study, minerals composition of the culture media was evaluated to verify the effect on cell wall permeability or buffering the medium and enzyme production. It is also known that the quantity and quality of nutrients available and the ability to assimilate successfully are the major determinants of microbial nature and its metabolic activity. The effects of minerals composition on lipolytic enzyme production by* Trichoderma *sp. F5,* T. harzianum *F37,* T. harzianum *F307,* Penicillium* sp. F36, and* F. solani *F61 were evaluated under submerged conditions, using olive oil as sole carbon source (Table 5). The highest lipase production was observed using medium M3, with 3.8-fold increase in production compared to the initial selection of fungus (F61 with 0.62 U/mL).* F. solani* F61 demonstrated hydrolytic activity of 2.37 U/mL and esterification activity of 0.07 U/mL. Yield fermentation (*Y*_*P/ S0*_) was 218.04 U/g of substrate. Medium M3 is composed of potassium, magnesium, phosphorus, copper, and sulfur. A sample under the same conditions, with no enzymatic extract present, was used as a control for each experiment. Pera et al. (2006) also developed this culture medium to produce lipases from* Aspergillus niger* under submerged conditions. Lipases from* Fusarium *spp. have some interesting properties, such as their stability in polar organic solvents like ethanol, acetone, and* n*-propanol. This characteristic is an important prerequisite for use in transesterification reactions in the presence of short-chain alcohols to produce biodiesel [43].

The medium composition strongly affected the lipase production of* F. solani *F61. The results observed for lipase production are directly related to the presence and concentration of each salt used in the media formulation. In this sense, the presence of calcium (CaCl_2_), zinc (ZnSO_4_), iron (FeSO_4_), and manganese (MnSO_4_) in the media negatively influenced enzyme production. On the other hand, for* Trichoderma *sp. F5,* Penicillium *sp. F36,* T. harzianum *F37, and* T. harzianum *F307, the highest lipase production was observed using medium 1 (M01) (0.42 – 0.73 U/mL), which is composed of MgSO_4_.H_2_O, K_2_HPO_4_, and CaCl_2_.2H_2_O. Esterification activity using this medium (M1) was only observed for* T. harzianum *F37 and F307. The highest esterification activity was observed with* T. harzianum *F307 (0.19 U/mL) using medium 5 (M5). Filamentous fungal strains presented different behaviors against the media composition indicating that the improved conditions for lipase production need to be optimized for each fungal strain in order to determine the ideal nutrient requirements of the cultures. In this regard, Sharma et al. (2001) reported that trace elements assist with structural components and are stabilizers of enzymes, whereas ions such as Mg^2+^, Fe^2+^, Ca^2+^, Cu^2+^, Co^2+^, Na^+^, K^+^, Mn^2+^, and Zn^2+^ appear to positively influence lipase production. The effect of the salts on the properties of the proteins is perceived by the activity, conformational stability, and solubility [44]. These effects may appear due to ionic bonds at specific sites of the proteins, altering their level of hydration [45]. Magnesium salt, for example, is required by most microorganisms because of their ability to perform some regulatory functions associated with increased metabolism [46]. Other micronutrients needed to regulate these functions are potassium, considered essential for osmoregulation [47], iron, which in turn is essentially used for synthesis of heme and cytochrome [48], and calcium, considered necessary for the effective stabilization of lipases [49]. These characteristics can be associated with structural changes of the enzyme and the fact that the salts increase the dispersion of enzymatic molecules, facilitating the mass transfer during the reaction [44].

### 3.4. Nitrogen Sources on Lipase Production

The effect of nitrogen sources on lipase production by* F. solani *F61 was carried out using the medium M3 supplemented with 1.0% (w/v) olive oil (Table 6). The concentrations of organic and inorganic nitrogen sources in the culture medium promoted an increased in lipase production; however maximum enzyme production was observed with 2.0% (w/v) tryptone (3.48 U/mL), followed by yeast extract at 1.0% and 2.0% (3.22 U/mL and 3.14 U/mL, respectively). Under these conditions, the highest biomass values (10.48 – 15.19 g/L) were also observed. Corn steep liquor and peptone provided intermediary values for lipase production, while urea, ammonium chloride, and ammonium sulfate provided the lowest values of enzyme production. It was observed that enzyme production was not associated with biomass formation, indicating that the nitrogen sources can modulate lipase secretion into the extracellular medium [50]. In this work, the parameters analyzed for* F. solani *F61 showed no* R*-Pearson correlation among the biomass and enzyme production (0.71) (Table 6). Fermentation parameters showed that yield of fermentation (*Y*_*P/ S0*_) with tryptone was 278.40 U/g; esterification activity was the highest with ammonium chloride (0.23 U/mL), followed by ammonium sulfate (0.17 U/mL) at 2.0% (w/v). Nitrogen sources, including organic nitrogen and inorganic nitrogen sources, play an important role in the synthesis of enzymes. As inorganic nitrogen sources can be used up quickly, organic nitrogen sources can supply many cell growth factors and amino acids, which are needed for cell metabolism and enzyme synthesis; therefore, both organic sources and inorganic sources are used in lipase fermentation [51]. Additionally, in the industrialization of fermentation processes, the nutrient cost of the medium is one of the most important factors, and supplementation of nitrogen source is too costly for commercial production of enzymes and biofuels, as it could amount to 50% of the overall medium cost [52].

### 3.5. Carbon Sources on Lipase Production

Several vegetable oils from what is considered “Legal Amazonian” were used as carbon sources as a substitute for olive oil. Media were prepared using tryptone and ammonium chloride at 2.0% (w/v) as nitrogen source (Table 7). Cotton oil was used as a carbon source by* F. solani *F61 and presented the highest lipase production value (8.14 U/mL) followed by soybean oil (6.17 U/mL) using tryptone as nitrogen source. Cotton oil is composed of palmitic acid (C16, 17-29%), stearic acid (C18, 1-5%), oleic acid (C18:1, 14-44%), and linoleic acid (C18:2, 33-58%), while soybean oil is composed of palmitic acid (C16, 2-13%), stearic acid (C18, 2-6%), oleic acid (C18:1, 18-31%), linoleic acid (C18:2, 49-57%), and linolenic acid (C18:3, 2-10%) [53]. The production of lipases in cotton and soybean oils may be related to oleic acid, as it is known to be an inducer of lipase production and is present in these oils in greater quantity than the other oils used. Furthermore, it may be responsible for higher enzymatic production, in addition to having been cited in literature as a good inducer of lipase production [54]. The parameters of fermentation for lipase production were* Y*_*P/ S0*_ = 732.60 U/g for cotton oil as a substrate and* Y*_*P/ S0*_ = 524.45 U/g for soybean oil. On the other hand, the usage of Amazonian oil with ammonium chloride as a nitrogen source presented the lowest value for lipase production (0.12 – 0.88 U/mL); however, the highest esterification activities were observed with copaiba oil (0.43 U/mL), babassu oil (0.41 U/mL), and castor oil (0.40 U/mL). Biomass production by* F. solani *F61 was also higher when the organic nitrogen (tryptone) with different Amazonian oils was used (8.9 - 14.5 g/L), while for the inorganic nitrogen source (ammonium chloride) the lowest biomass production was observed (1.7 – 8.6 g/L). Although copaiba oil has a large amount of oleic acid, the enzymatic production of lipase, as well as its biomass production, was not high, showing that high amounts of oleic acid concentrations may also inhibit lipase production [55]. The biomass produced and the hydrolytic activity for each carbon source showed no correlation* R-*Pearson (0.67 for tryptone and -0.03 for ammonium chloride, respectively).

### 3.6. Time-Course of Lipase Production

**Figure 1:**
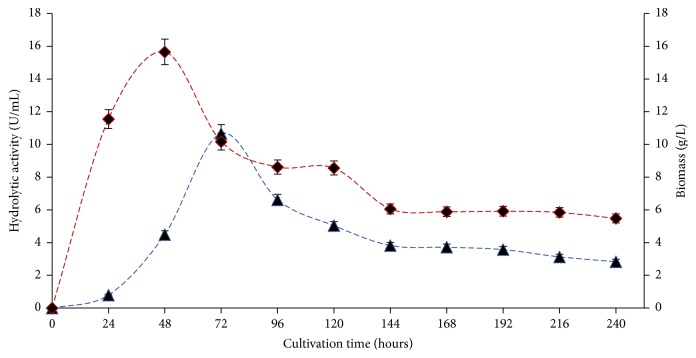
Time-course for lipolytic enzyme production and biomass of* F. solani*.F61. (▲ hydrolytic activity and ♦ biomass).

*F. solani* F61 was cultured for 240 hours to verify the time-course for lipase production using tryptone 2.0% (w/v) and cotton oil 1.0% (w/v), 28°C and 180 rpm (Figure 1). In the first 24 hours of cultivation, lipase production was observed at 0.80 U/mL, with continuous increase up to 72 hours of incubation (10.68 U/mL), and the yield of fermentation was 1,158.00 U/g of substrate. After this period, the enzyme production decreased continuously up to 240 hours. Winaynuwattikun et al. (2011) evaluated the time-course for lipase production of an* F. solani* strain, and the maximum enzyme production was observed after 72 hours (1.26 U/mL) during the initial stationary phase. Salihu et al. (2011) evaluated the lipase production of several microorganisms isolated from palm oil factory effluents. In that study,* C. cylindracea *was observed to demonstrate maximum enzyme activity after 144 h (2.1 U/mL). The decrease in enzyme production is associated the extended cultivation period, nutrient depletion, or protease activity. Mahanta et al. (2008) observed these conditions during the solid-state cultivation of* Pseudomonas aeruginosa* in that the decrease in enzyme activity was associated with nutrient depletion or denaturation of the enzyme caused by interaction with other components in the environment or alteration in the pH of the medium. Long et al. (2007) reported that several factors are known to influence the expression of lipase activity, one of them being component of the medium, such as carbon sources, nitrogen, as well as inducers in the form of oils, fatty acids, and esters. These factors are known to influence the production of lipases.

## 4. Concluding Remarks

The selection of lipolytic filamentous fungi isolated from senescent plant material in Cerrado streams showed that, even under conditions not suitable for the induction of lipases, it is possible to find microorganisms with the potential for enzymatic production. The vegetation present in this natural habitat is inductive of a potential energy source for microbe's growth and enzyme production. The potential of the numerous wild strains reported here to produce lipases using unconventional oils is evidenced in this work. The basal composition of the culture medium was essential to increase lipase production, and its supplementation with tryptone (2.0% w/v) and degummed cottonseed oil (1.0% w/v) increased the production 13.3-fold (8.14 U/mL). The growth curve of the fungus showed that the highest production of lipases was observed after 72 hours of incubation (10.68 U/mL). Under these conditions, there was a 17.22-fold increase in enzyme production. The enzyme produced by* F. solani* F61 showed potential to be used in hydrolysis and transesterification processes in organic media to produce fatty acid esters. An in-depth study into their production and activity through optimization processes is suggested, as it would provide a better understanding of this valuable enzyme.

## Figures and Tables

**Table 1:** Medium of composition for lipolytic enzymes production.

Medium	Mineral formulation (g/L)	Reference
(M1)	MgSO_4_.H_2_O 0.14; K_2_HPO_4_ 0.50; CaCl_2_.2H_2_O 7.20;	[21]
(M2)	NaH_2_PO_4_ 12.00; K_2_HPO_4_ 2.00; CaCl_2_.2H_2_O 0.03; ZnSO_4_.H_2_O 0.03; FeSO_4_.7H_2_O 0.005;	[22]
(M3)	KH_2_PO_4_ 1,00; MgSO_4_.H_2_O 1,123; CuSO_4_ 0.06;	[23]
(M4)	MgSO_4_.H_2_O 0.28; KCl 0,50; K_2_HPO_4_ 2.00; FeSO_4_.7H_2_O 0.005; NaH_2_PO_4_ 2.00;	[24]
(M5)	KH_2_PO_4_ 1.00; MgSO_4_.H_2_O 0.28; CaCl_2_.2H_2_O 0.13; NaCl 0.10; H_3_BO_3_ 0.0005; CuSO_4_.5H_2_O 0.00004; KI 0.00001; FeCl_3_.6H_2_O 0.00023; ZnSO_4_.7H_2_O 0.0004; MnSO_4_.H_2_O 0.0004	[25]
(M6)	MgSO_4_.H_2_O 0.28; ZnSO_4_.7H_2_O 0.40; FeSO_4_.7H_2_O 1.83; MnSO_4_.H_2_O 0.12	[26]

**Table 2:** Screening of filamentous fungi in solid medium using Rhodamine B and Tween 20.

Strains	Rhodamine B	Tween 20	Enzymatic activity ^1^
F2	-	-	n.d
F3	+	+	+
F4	-	-	n.d
F5	+	+	+
F7	+	+	+
F8	-	-	n.d
F11	-	-	n.d
F14	+	+	+
F15	+	+	+
F16	+	+	+
F20	+	+	+
F21	+	+	+
F22	+	+	+
F23	-	-	n.d
F24	+	+	+
F25	-	-	n.d
F26	+	+	+
F28	-	-	n.d
F29	-	-	n.d
F30	+	+	+
F31	+	+	+
F32	+	+	+
F33	+	+	+
F35	+	+	+
F36	+	+	+
F37	+	+	+
F59	-	-	n.d
F61	+	+	+
F77	-	-	n.d
F85	-	-	n.d
F91	-	-	n.d
F104	+	+	+
F116	+	+	+
F120	+	+	+
F125	+	+	+
F130	+	+	+
F137	-	-	n.d
F307	+	+	+

Culture conditions: ^1^ agar plate incubation was carried out with Rhodamine B (olive oil) and Tween 20 at 28°C for 7 days. Label: (+) presence of activity, (-) absence of activity; n.d. no activity detected under the assay conditions.

**Table 3:** Screening of filamentous fungi for lipolytic enzymes production with hydrolytic and esterification activities.

Strains	Biomass (g/L)	Hydrolytic activity (U/mL) ^1^	Esterification activity (U/mL)
F2	-	-	-
F3	4.25 ± 0.22 i ^2^	0.05 ± 0.01 f	n.d.
F4	-	-	-
F5	3.79 ± 0.35 j	0.07 ± 0.02 e	0.09 ± 0.04 b
F7	3.89 ± 0.04 j	0.04 ± 0.01 f	n.d.
F8	-	-	-
F11	-	-	-
F14	3.44 ± 0.07 k	0.03 ± 0.00 f	n.d.
F15	3.42 ± 0.03 k	0.02 ± 0.01 f	n.d.
F16	2.40 ± 0.02 l	0.08 ± 0.02 e	n.d.
F20	1.66 ± 0.12 m	0.03 ± 0.003 f	n.d.
F21	0.94 ± 0.06 n	0.01 ± 0.00 f	n.d.
F22	9.19 ± 0.56 d	0.25 ± 0.04 b	0.02 ± 0.01 f
F23	-	-	-
F24	10.76 ± 0.03 a	0.07 ± 0.00 e	n.d.
F25	-	-	-
F26	9.51 ± 0.01 c	0.06 ± 0.00 e	n.d.
F28	-	-	-
F29	-	-	-
F30	7.00 ± 0.15 g	0.07 ± 0.01 e	n.d.
F31	9.40 ± 0.12 c	0.19 ± 0.04 c	0.0
F32	8.91 ± 0.57 e	0.08 ± 0.00 e	n.d.
F33	8.39 ± 0.16 f	0.12 ± 0.02 d	0.06 ± 0.02 d
F35	4.06 ± 0.06 i	0.12 ± 0.01 d	n.d.
F36	8.54 ± 0.06 f	0.19 ± 0.06 c	0.11 ± 0.05 a
F37	8.76 ± 0.02 e	0.19 ± 0.05 c	0.08 ± 0.04 c
F59	-	-	-
F61	5.12 ± 0.04 h	0.62 ± 0.02 a	0.09 ± 0.04 b
F77	-	-	-
F85	-	-	-
F91	-	-	-
F104	8.51 ± 0.06 f	0.18 ± 0.07 c	0.04 ± 0.02 e
F116	9.14 ± 0.03 d	0.06 ± 0.007 e	n.d.
F120	10.14 ± 0.08 b	0.05 ± 0.00 f	n.d.
F125	9.68 ± 0.05 c	0.05 ± 0.01 f	n.d.
F130	9.08 ± 0.09 d	0.07 ± 0.00 e	n.d.
F137	-	-	-
F307	9.68 ± 0.01 c	0.16 ± 0.02 c	0.10 ± 0.04 a

Culture conditions: ^1^ Submerged cultivation was carried out in Vogel medium with 1.0% olive oil and 0.2% (w/v) yeast extract at 28°C, 180 rpm for 5 days. Label: (+) presence of activity, (-) absence of activity; n.d. no activity detected under the assay conditions. ^2^ Statistically significant differences between the mean values of hydrolytic activity and esterification were defined using the Scott-Knott test. Averages followed by distinct letters differ by Scott-Knott test at the level of significance 0.05. Pearson correlation was determined using biomass and hydrolytic activity. *R*-Pearson correlation: 0.16.

**Table 4:** Molecular identification of filamentous fungi producing lipase.

Strains	Code UFT	Species	Best Match Database	Similarity index (%)	accession numbers
			(GenBank Accession No.)		ITS
F61	8906	*Fusarium solani*	KT184398.1	99	MK649917
F03	8907	*Aspergillus calidoustus*	HG931696.1	99	MK649918
F21	8908	*Chaetomium aureum*	GU966501.1	99	MK649919
F37	8909	*Trichoderma harzianum*	KC342029.1	99	MK649920
F104	8910	*Aspergillus flavus*	KF221065.1	99	MK649921
F116	8911	*Aspergillus flavus*	KX270348.1	99	MK649922
F120	8912	*Penicillium citrinum*	LC106114.1	99	MK649923
F125	8913	*Aspergillus *sp	KP721583.1	99	MK649924
F130	8914	*Aspergillus oryzae*	KJ150716.1	98	MK649925
F307	8915	*Trichoderma harzianum*	KJ028794.1	99	MK649926

**Table 5:** Lipolytic enzyme production with hydrolytic and esterification activities in different media composition.

Strains	Media compositions	Hydrolytic activity (U/mL)	Y_P/S0_ (U/g substrate)	Esterification activity (U/mL)
*Trichoderma* sp. F5	M1	0.71 ± 0.01 b ^1^	59.64	n.d
	M2	0.06 ± 0.01 f	5.04	0.05 ± 0.01 e
	M3	0.03 ± 0.00 f	2.40	0.03 ± 0.01 f
	M4	0.05 ± 0.00 f	4.00	n.d
	M5	0.03 ± 0.00 f	2.52	n.d
	M6	0.03 ± 0.00 f	2.52	n.d
				
*Penicillium* sp. F36	M1	0.63 ± 0.00 b	52.92	n.d
	M2	0.24 ± 0.00 d	22.08	n.d
	M3	0.10 ± 0.01 f	8.00	n.d
	M4	0.18 ± 0.00 e	15.12	n.d
	M5	0.19 ± 0.00 e	17.48	0.04 ± 0.01 f
	M6	0.02 ± 0.00 f	1.76	0.09 ± 0.05 b
				
*T. harzianum* F37	M1	0.42 ± 0.01 c	36.96	0.03 ± 0.01 f
	M2	0.02 ± 0.01 f	1.68	0.08 ± 0.00 c
	M3	0.16 ± 0.04 e	15.36	n.d
	M4	0.10 ± 0.02 f	8.80	n.d
	M5	0.24 ± 0.03 d	20.16	n.d
	M6	0.08 ± 0.00 f	7.04	0.05 ± 0.01 e
				
*F. solani* F61	M1	0.73 ± 0.01 b	67.16	n.d
	M2	0.35 ± 0.01 c	32.2	n.d
	M3	2.37 ± 0.01 a	218.04	0.07 ± 0.01 d
	M4	0.45 ± 0.02 c	46.06	0.03 ± 0.00 f
	M5	0.65 ± 0.01 b	57.20	0.01 ± 0.00 f
	M6	0.02 ± 0.00 f	1.84	n.d
				
*T. harzianum* F307	M1	0.42 ± 0.01 c	40.32	0.05 ± 0.01 e
	M2	0.03 ± 0.00 f	2.88	0.18 ± 0.00 a
	M3	0.06 ± 0.00 f	5.76	0.05 ± 0.01 e
	M4	0.04 ± 0.00 f	3.52	n.d
	M5	0.24 ± 0.01 d	23.04	0.19 ± 0.00 a
	M6	0.04 ± 0.00 f	3.36	0.02 ± 0.00 f
				

Culture conditions: cultivation was carried out with 1.0% olive oil and 0,2% yeast extract, at 28°C and 180 rpm for 5 days. A sample under the same conditions was used as control for each experiment. Label: n.d. no activity detected under the assay conditions. Statistically significant differences between the mean values of biomass, hydrolytic activity, and esterification were defined using the Scott-Knott test. ^1^averages followed by distinct letters differ by Scott-Knott test at the level of significance 0.05.

**Table 6:** Nitrogen sources for lipolytic enzyme production with hydrolytic and esterification activities for *F. solani* (F61).

Nitrogen sources	Concentration (%)	Biomass (g/L)	Hydrolytic activity (U/mL)	*Y*_(*P*/*S*0)_ (U/g)	Esterification activity (U/mL)
Organic sources					
Corn steep liquor	0.2	5.52 ± 0.01 o ^1^	0.05 ± 0.01 p	5.25	n.d
	1.0	7.54 ± 0.04 j	0.49 ± 0.01 k	44.10	n.d
	2.0	9.45 ± 0.04 e	1.10 ± 0.11 h	71.50	n.d
Peptone	0.2	6.86 ± 0.02 l	0.21 ± 0.02 m	22.05	n.d
	1.0	8.21 ± 0.02 g	2.15 ± 0.02 f	204.25	0.04 ± 0.01 d
	2.0	9.43 ± 0.02 f	1.34 ± 0.14 g	127.30	n.d
Tryptone	0.2	4.45 ± 0.01 p	0.96 ± 0.02 i	91.20	n.d
	1.0	10.48 ± 0.02d	2.89 ± 0.01 d	231.30	0.05 ± 0.01 d
	2.0	15.19 ± 0.01 a	3.48 ± 0.01 a	278.40	0.02 ± 0.00 e
Urea	0.2	6.54 ± 0.02 n	0.11 ± 0.02 o	11.50	0.01 ± 0.00 e
	1.0	7.72 ± 0.02 h	0.41 ± 0.02 l	45.10	n.d
	2.0	7.64 ± 0.02 i	0.57 ± 0.04 j	62.70	n.d
Yeast extract	0.2	3.18 ± 0.01 r	2.37 ± 0.02 e	237.00	0.03 ± 0.01 e
	1.0	10.88 ± 0.01 c	3.22 ± 0.07 b	241.50	0.03 ± 0.01 e
	2.0	14.85 ± 0.01 b	3.14 ± 0.02 c	251.20	n.d

Inorganic sources					
Ammonium chloride	0.2	3.54 ± 0.02 q	0.05 ± 0.01 p	5.00	n.d
	1.0	0.10 ± 0.01 u	0.10 ± 0.01 o	10.50	0.06 ± 0.01 d
	2.0	0.17 ± 0.01 t	0.16 ± 0.02 n	17.60	0.23 ± 0.02 a
Ammonium sulfate	0.2	7.06 ± 0.02 k	0.06 ± 0.01 p	6.00	0.02 ± 0.01 e
	1.0	3.01 ± 0.02 s	0.11 ± 0.01 o	12.10	0.10 ± 0.01 c
	2.0	6.78 ± 0.01 m	0.21 ± 0.01 m	24.15	0.17 ± 0.01 b

Culture conditions: submerged cultivation was carried out in medium containing g/l - KH_2_PO_4_ – 1, MgSO_4_.H_2_O – 1.123 e CuSO_4_ – 0.06. Supplemented with 1.0% olive oil at 28°C, 180 rpm for 5 days. Label: n.d. no activity detected under the assay conditions. ^1^averages followed by distinct letters differ by Scott-Knott test at the level of significance 0.05. Pearson correlation was determined using biomass and hydrolytic activity. *R*-Pearson correlation: 0.71.

**Table 7:** Effect of vegetable oils on the lipase production of *F. solani* F61 using tryptone or ammonium chloride as a nitrogen source.

Carbon sources	Concentration (%)	Biomass (g/L)	Hydrolytic activity (U/mL)	*Y*_(*P*/*S*0)_ (U/g)	Esterification activity (U/mL)
*Tryptone*					
Castor oil	1	13.99 ± 0.02 b ^1^	3.77 ± 0.01 e	339.30	0.09 ± 0.01 c
Pequi Oil	1	13.6 ± 0.02 d	2.88 ± 0.01 g	273.60	0.10 ± 0.00 b
Babassu Oil	1	14.5 ± 0.05 a	3.50 ± 0.01 f	192.50	0.03 ± 0.00 f
Copaiba Oil	1	8.98 ± 0.03 g	0.29 ± 0.01 h	29.00	0.07 ± 0.01 d
Buriti oil	1	13.14 ± 0.02 e	4.51 ± 0.01 d	451.00	0.14 ± 0.00 a
Degummed Soybean Oil	1	13.95 ± 0.03 c	6.17 ± 0.01 b	524.45	0.07 ± 0.00 d
Cotton Oil Degomado	1	13.94 ± 0.02 c	8.14 ± 0.01 a	732.60	0.05 ± 0.00 e
Frying Oil	1	13.00 ± 0.04 f	5.02 ± 0.01 c	351.40	0.05 ± 0.00 e
*Ammonium chloride*					
Castor oil	1	8.64 ± 0.02 a	0.18 ± 0.01 d	19.80	0.40 ± 0.03 c
Pequi Oil	1	5.70 ± 0.04 c	0.12 ± 0.01 f	14.40	0.30 ± 0.02 e
Babassu Oil	1	4.59 ± 0.02 e	0.88 ± 0.01 a	105.60	0.41 ± 0.02 b
Copaiba Oil	1	3.03 ± 0.01 g	0.12 ± 0.01 f	13.80	0.43 ± 0.01 a
Buriti oil	1	5.98 ± 0.01 b	0.24 ± 0.01 c	27.60	0.36 ± 0.01 d
Degummed Soybean Oil	1	5.10 ± 0.01 d	0.14 ± 0.01 e	14.00	0.36 ± 0.01 d
Cotton Oil Degomado	1	4.02 ± 0.01 f	0.39 ± 0.01 b	42.90	0.28 ± 0.02 g
Frying Oil	1	1.74 ± 0.01 h	0.14 ± 0.01 e	16.10	0.29 ± 0.01 f

Culture conditions: submerged cultivation was carried out in medium containing g/L- KH_2_PO_4_ – 1, MgSO_4_.H_2_O – 1.123 e CuSO_4_ – 0.06. Supplemented with 2.0% Tryptone and 2.0% Ammonium chloride at 28°C, 180 rpm for 5 days. Label: n.d. no activity detected under the assay conditions. ^1^averages followed by distinct letters differ by Scott-Knott test at the level of significance 0.05. Pearson correlation was determined using biomass and hydrolytic activity. *R*-Pearson correlation: 0.67 for Tryptone 2.0% and *R*-Pearson correlation: -0.03 for Ammonium chloride 2.0%.

## Data Availability

The data obtained as a result of this research were obtained from microorganisms deposited in the Carlos Augusto Rosa cultures collection of the Federal University of Tocantins, which are available for future studies.

## References

[1] Gupta R., Gupta N., Rathi P. (2004). Bacterial lipases: An overview of production, purification and biochemical properties. *Applied Microbiology and Biotechnology*.

[2] Praveen Kumar P., Sagaya Jansi R., Saravana Kumar P. (2017). Optimization of biosynthesis parameters, partial purification and characterization of extracellular lipase from soil derived Streptomyces sp. Loyola Lipase-1. *Biocatalysis and Agricultural Biotechnology*.

[3] Kumar A., Dhar K., Kanwar S. S., Arora P. K. (2016). Lipase catalysis in organic solvents: Advantages and applications. *Biological Procedures Online*.

[4] Kuperkar V. V., Lade V. G., Prakash A., Rathod V. K. (2014). Synthesis of isobutyl propionate using immobilized lipase in a solvent free system: optimization and kinetic studies. *Journal of Molecular Catalysis B: Enzymatic*.

[5] Tacias-Pascacio V. G., Virgen-Ortíz J. J., Jiménez-Pérez M. (2017). Evaluation of different lipase biocatalysts in the production of biodiesel from used cooking oil: Critical role of the immobilization support. *Fuel*.

[6] Gutarra M. L. E., Godoy M. G., Maugeri F., Rodrigues M. I., Freire D. M. G., Castilho L. R. (2009). Production of an acidic and thermostable lipase of the mesophilic fungus Penicillium simplicissimum by solid-state fermentation. *Bioresource Technology*.

[7] Rodrigues C., Cassini S. T., Antunes P. W., Keller R. P., Gonçalves R. F. (2016). Isolation and selection of lipase-producing fungi based on lipase activity and hydrolytic potential on soybean oil and grease trap scum. *Engenharia Sanitária e Ambiental*.

[8] Pandey A. (2003). Solid-state fermentation. *Biochemical Engineering Journal*.

[9] Hasan F., Shah A. A., Hameed A. (2009). Methods for detection and characterization of lipases: A comprehensive review. *Biotechnology Advances*.

[10] Hankin L., Anagnostakis S. L. (1975). The use of solid media for detection of enzyme production by fungi. *Mycologia*.

[11] Colen G. (2006). *Isolamento e seleção de fungos filamentosos produtores de lipases*.

[12] Nobre F. S. (2012). *Lipase activity and biodiversity of filamentous fungi derived from antarctica. Dissertação de mestrado*.

[13] Canseco-Pérez M., Castillo-Avila G., Chi-Manzanero B. (2018). Fungal screening on olive oil for extracellular triacylglycerol lipases: selection of a trichoderma harzianum strain and genome wide search for the genes. *Gene*.

[14] Roveda M., Hemkemeier M., Colla L. M. (2010). Evaluation of lipase production using different strains of microorganisms isolated from dairy effluent through submerged fermentation. *Food Science and Technology*.

[15] Salihu A., Alam M. Z., AbdulKarim M. I., Salleh H. M. (2011). Suitability of using palm oil mill effluent as a medium for lipase production. *African Journal of Biotechnology*.

[16] Cortez D. V., Castro H. F. de., Andrade G. S. S. (2017). Potential catalytic of mycelium-bound lipase of filamentous fungi in biotransformation processes. *Quim. Nova*.

[17] Penha E. d., Viana L. d., Gottschalk L. M. (2016). Aproveitamento de resíduos da agroindústria do óleo de dendê para a produção de lipase por Aspergillus Níger. *Ciência Rural , Santa Maria*.

[18] Ribeiro dos Santos R., Nolasco Macedo Muruci L., Oliveira Santos L., Antoniassi R., Passos Lima da Silva J., Caramez Triches Damaso M. (2014). Characterization of Different Oil Soapstocks and Their Application in the Lipase Production by Aspergillus niger under Solid State Fermentation. *Journal of Food and Nutrition Research*.

[19] Toscano L., Montero G., Stoytcheva M. (2013). Lipase production through solid-state fermentation using agro-industrial residues as substrates and newly isolated fungal strains. *Biotechnology & Biotechnological Equipment*.

[20] Ribeiro E., Silva G., Kamimura E. S., Aguiar Oliveira E., Resende Maldonado R. Produção seletiva de lipase por geotrichum candidum em fermentação submersa, utilizando óleo de algodão: otimização e caracterização de especificidade. https://proceedings.science/cobeq/cobeq-2016/papers/producao-seletiva-de-lipase-por-geotrichum-candidum-em-fermentacao-submersa%2C-utilizando-oleo-de-algodao%3A-otimizacao-e-ca.

[21] Kashmiri M. A., Adnan A., Butt B. W. (2006). Production, purification and partial characterization of lipase from Trichoderma Viride. *African Journal of Biotechnology*.

[22] Ulker S., Ozel A., Colak A., Karaoglu A. S., Karaoğlu A. Ş. (2011). Isolation, production, and characterization of an extracellular lipase from Trichoderma harzianum isolated from soil. *Turkish Journal of Biology*.

[23] Pera L. M., Romero C. M., Baigori M. D., Castro G. R. (2006). Catalytic properties of lipase extracts from aspergillus niger. *Food Technology and Biotechnology*.

[24] Adham N. Z., Ahmed E. M. (2009). Extracellular lipase of Aspergillus niger NRRL3; production, partial purification and properties. *Indian Journal of Microbiology*.

[25] Olson B. H., Johnson M. J. (1948). Factors producing high yeast yields in synthetic media. *Journal of Bacteriology*.

[26] Gochev V., Montero G., Kostov G. (2012). Nutritive medium engineering enhanced production of extracellular lipase by trichoderma longibrachiatum. *Biotechnology & Biotechnological Equipment*.

[27] Kearse M., Moir R., Wilson A. (2012). Geneious Basic: an integrated and extendable desktop software platform for the organization and analysis of sequence data. *Bioinformatics*.

[28] Altschul S. F., Gish W., Miller W., Myers E. W., Lipman D. J. (1990). Basic local alignment search tool. *Journal of Molecular Biology*.

[29] Kumar D., Kumar L., Nagar S., Raina C., Parshad R., Gupta V. K. (2012). Screening, isolation and production of lipase/esterase producing Bacillus sp. strain DVL2 and its potential evaluation in esterification and resolution reactions. *Archives of Applied Science Research*.

[30] Vogel H. J. (1956). A convenient growth medium for Neurospora crassa (medium N). *Microbiology Genetics Bulletin*.

[31] De Almeida A. F., Taulk-Tornisielo S. M., Carmona E. C. (2013). Influence of carbon and nitrogen sources on lipase production by a newly isolated Candida viswanathii strain. *Annals of Microbiology*.

[32] Rigo E., Polloni A. E., Remonatto D. (2010). Esterification activity of novel fungal and yeast lipases. *Applied Biochemistry and Biotechnology*.

[33] Filho D. B. F., Júnior J. A. S. (2009). *Desvendando os Mistérios do Coeficiente de Correlação de Pearson (r)*.

[34] Geoffry K., Achur R. N. (2018). Screening and production of lipase from fungal organisms. *Biocatalysis and Agricultural Biotechnology*.

[35] Bentubo H. D. L., Gompertz O. F. (2014). Effects of temperature and incubation time on the in vitro expression of proteases, phospholipases, lipases and DNases by different species of Trichosporon. *SpringerPlus*.

[36] Naz S., Jadhav S. K. (2015). Studies of the estimation of lipase production capability of some fungal species and their application in oil spillage degradation. *International Journal of Science and Research (IJSR)*.

[37] Sadati R., Barghi A., abbasi Larki R. (2015). Isolation and Screening of Lipolytic Fungi From Coastal Waters of the Southern Caspian Sea (North of Iran). *Jundishapur Journal of Microbiology*.

[38] Schoch C. L., Seifert K. A., Huhndorf S. (2012). Nuclear ribosomal internal transcribed spacer (ITS) region as a universal DNA barcode marker for *Fungi*. *Proceedings of the National Acadamy of Sciences of the United States of America*.

[39] Dos Santos T. T., De Souza Leite T., De Queiroz C. B., de Araújo E. F., Pereira O. L., De Queiroz M. V. (2016). High genetic variability in endophytic fungi from the genus Diaporthe isolated from common bean (Phaseolus vulgaris L.) in Brazil. *Journal of Applied Microbiology*.

[40] Soares D. A., De Oliveira D. P., Dos Santos T. T., Marson P. G., Pimenta R. S. (2018). Multiloci identification of Diaporthe fungi isolated from the medicinal plant Costus spiralis (Jacq.) Roscoe (Costaceae). *Journal of Applied Microbiology*.

[41] Liu Y., Leigh J. W., Brinkmann H. (2008). Phylogenomic Analyses Support the Monophyly of Taphrinomycotina, including Schizosaccharomyces Fission Yeasts. *Molecular Biology and Evolution*.

[42] Long Z., Xu J., Pan J. (2007). Significant improvement of serratia marcescens lipase fermentation, by optimizing medium, induction, and oxygen supply. *Applied Biochemistry and Biotechnology*.

[43] Oliveira B. H., Coradi G. V., Attili-Angelis D. (2013). Comparison of lipase production on crambe oil and meal by fusarium sp. (gibberella fujikuroi complex). *European Journal of Lipid Science and Technology*.

[44] Pacheco S. M., Júnior A. C., Morgado A. F. (2015). Isolation and screening of filamentous fungi producing extracellular lipase with potential in biodiesel production. *Advances in Enzyme Research*.

[45] Yu H. W., Chen H., Yang Y. Y., Ching C. B. (2005). Effect of salts on activity, stability and enantioselectivity of Candida rugosa lipase in isooctane. *Journal of Molecular Catalysis B: Enzymatic*.

[46] Bankar S. B., Bule M. V., Singhal R. S., Ananthanarayan L. (2009). Optimization of Aspergillus niger fermentation for the production of glucose oxidase. *Food and Bioprocess Technology*.

[47] Salihu A., Alam Z., Alam Md. Z. (2012). Production and applications of microbial lipases: A review. *Scientific Research and Essays*.

[48] Venkateshwar M., Chaitanya K., Altaf M., Mahammad E. J., Bee H., Reddy G. (2010). Influence of micronutrients on yeast growth and *β*-d-fructofuranosidase production. *Indian Journal of Microbiology*.

[49] Snellman E. A., Colwell R. R. (2004). Acinetobacter lipases: Molecular biology, biochemical properties and biotechnological potential. *Journal of Industrial Microbiology and Biotechnology*.

[50] Fickers P., Nicaud J., Gaillardin C., Destain J., Thonart P. (2004). Carbon and nitrogen sources modulate lipase production in the yeast Yarrowia lipolytica. *Journal of Applied Microbiology*.

[51] Tan T., Zhang M., Xu J., Zhang J. (2004). Optimization of culture conditions and properties of lipase from *Penicillium camembertii Thom* PG-3. *Process Biochemistry*.

[52] Edwinoliver N. G., Thirunavukarasu K., Purushothaman S., Rose C., Gowthaman M. K., Kamini N. R. (2009). Corn steep liquor as a nutrition adjunct for the production of Aspergillus niger lipase and hydrolysis of oils thereof. *Journal of Agricultural and Food Chemistry*.

[53] Yang A., Qi M., Wang X. (2019). Refined cottonseed oil as a replacement for soybean oil in broiler diet. *Food Science & Nutrition*.

[54] Li D., Wang B., Tan T. (2006). Production enhancement of Rhizopus arrhizus lipase by feeding oleic acid. *Journal of Molecular Catalysis B: Enzymatic*.

[55] Wang D., Xu Y., Shan T. (2008). Effects of oils and oil-related substrates on the synthetic activity of membrane-bound lipase from *Rhizopus chinensis* and optimization of the lipase fermentation media. *Biochemical Engineering Journal*.

